# A quick guide for using Microsoft OneNote as an electronic laboratory notebook

**DOI:** 10.1371/journal.pcbi.1006918

**Published:** 2019-05-09

**Authors:** Santiago Guerrero, Andrés López-Cortés, Jennyfer M. García-Cárdenas, Pablo Saa, Alberto Indacochea, Isaac Armendáriz-Castillo, Ana Karina Zambrano, Verónica Yumiceba, Andy Pérez-Villa, Patricia Guevara-Ramírez, Oswaldo Moscoso-Zea, Joel Paredes, Paola E. Leone, César Paz-y-Miño

**Affiliations:** 1 Centro de Investigación Genética y Genómica, Facultad de Ciencias de la Salud Eugenio Espejo, Universidad UTE, Quito, Ecuador; 2 RNASA-IMEDIR, Computer Sciences Faculty, University of Coruna, Coruna, Spain; 3 Faculty of Engineering Sciences, Universidad UTE, Quito, Ecuador; 4 Gene Regulation, Stem Cells and Cancer Programme, Centre for Genomic Regulation (CRG), The Barcelona Institute of Science and Technology, Barcelona, Spain; 5 Oncology and Molecular Pathology Research Group-VHIR-Vall d’ Hebron Institut de Recerca-Vall d’ Hebron Hospital, Barcelona, Spain; University of Toronto, CANADA

## Abstract

Scientific data recording and reporting systems are of a great interest for endorsing reproducibility and transparency practices among the scientific community. Current research generates large datasets that can no longer be documented using paper lab notebooks (PLNs). In this regard, electronic laboratory notebooks (ELNs) could be a promising solution to replace PLNs and promote scientific reproducibility and transparency. We previously analyzed five ELNs and performed two survey-based studies to implement an ELN in a biomedical research institute. Among the ELNs tested, we found that Microsoft OneNote presents numerous features related to ELN best functionalities. In addition, both surveyed groups preferred OneNote over a scientifically designed ELN (PerkinElmer Elements). However, OneNote remains a general note-taking application and has not been designed for scientific purposes. We therefore provide a quick guide to adapt OneNote to an ELN workflow that can also be adjusted to other nonscientific ELNs.

This is a *PLOS Computational Biology* Education paper.

## Introduction

Scientific data recording and reporting systems are of great interest for endorsing reproducibility and transparency practices among the scientific community [1–3]. Current experimental [4–6] and bioinformatic research [7] generates large datasets or high-resolution images [8] that can no longer be documented using paper lab notebooks (PLNs). In this regard, electronic laboratory notebooks (ELNs), which are gradually replacing PLNs in academic and pharmaceutical research [9–12], could be a promising solution to achieve good documentation practices and promote scientific reproducibility and transparency.

ELN development has increased notably during the last few years, from commercial solutions to open-source software. Kanza and colleagues [3] identified 72 active ELNs specialized either in specific disciplines or in all-purpose solutions. Compared with PLNs, ELNs could improve data acquisition, archiving, accessibility, sharing, and even data presentation during personal and lab meetings [3,12].

We previously analyzed five ELNs using 42 parameters related to ELN best functionalities [12]. Among the ELNs tested, we found that Microsoft OneNote presents almost all parameters evaluated (39/42). We also performed two survey-based studies among 28 scientists and 80 students to evaluate OneNote performance compared with a scientifically designed ELN (PerkinElmer Elements) [12]. Although OneNote is not an ELN *per se*, both surveyed groups preferred OneNote as an ELN [12]. Indeed, OneNote remains a general note-taking application and has not been designed for scientific purposes. We therefore provide a quick guide to adapt OneNote to an ELN workflow that can also be adjusted to other nonscientific ELNs.

## Structure and labeling

**Fig 1:**
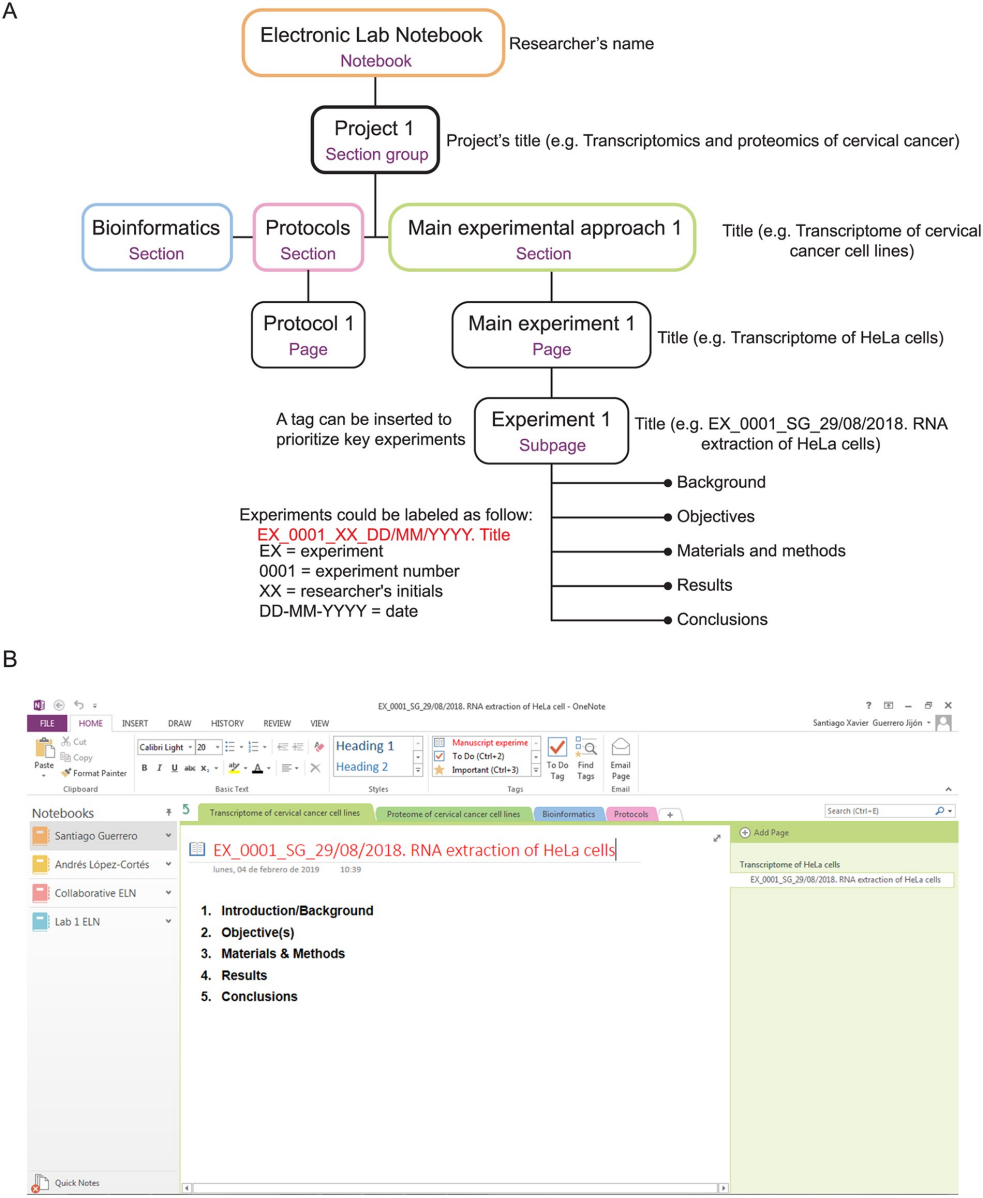
Adaptation of Microsoft OneNote’s hierarchical structure to an ELN workflow. (A) The structure of OneNote (violet) and its adaptation to a scientific setting is presented. (B) A screenshot of a OneNote ELN hierarchical structure is shown. ELN, electronic lab notebook.

OneNote provides a hierarchical structure that can be adapted to an ELN workflow. Based on this organization, a notebook can encompass unlimited projects (Section Groups in OneNote). A project can contain unlimited sections (e.g., protocols); meanwhile, experiments can be arranged using at least three hierarchical layers: sections, pages, and subpages (Fig 1). An experiment or any other analysis should be structured using five essential parts: (1) an introduction to the experiment describing, for example, a hypothesis to be tested; (2) a detailed description of the objective(s) of the experiment; (3) a materials and methods section providing all materials or reagents along with their references or lot numbers (specific methods or protocols could be hyperlinked to this part [Insert a link feature or Crtl+K in OneNote]); (4) a results part presenting all main outputs of the experimental approach; and (5) conclusions discussing the main findings and recommendations for further research. To facilitate ELN usage, OneNote allows users to create templates that can contain the aforementioned five elements or specific protocols (e.g., PCRs). Customizable tags can also be used to prioritize or organize experiments or any other entry. For instance, a customizable tag can be applied to easily find and recognize key experiments or protocols important for constructing a manuscript.

Nowadays, scientific laboratories accumulate a large amount of data that is of varying quality and usefulness. OneNote’s searching functionality allows users to quickly retrieve important information through this vast amount of data. Thus, experiments, protocols, or any other entry should be labeled with essential information traceable over time. For example, an experiment can be named *EX_0001_SG_29/08/2018*. *RNA extraction of HeLa cells*, in which *EX* = experiment, *0001* = experiment number, *SG* = researcher’s initials, *29/08/2018* = entry date, and *RNA extraction of HeLa cells* = title of the experiment. Moreover, experiments, protocols, or bioinformatic analyses could be labeled accordingly: experiments = *EX*, protocols = *PR*, and bioinformatics = *BI* (Fig 1). To improve labeling, you can also check, “Ten simple rules for experiments’ provenance” by Kazic [13].

## Data acquisition

All data resulting from research experiments, analyses, and observations must be recorded without exception. Details from *in silico* analyses, bioinformatic pipelines, scripts, or any other computational-related codes or methodologies could be recorded too [14,15]. As previously discussed within PLOS’s Ten Simple Rules collection, Schnell [16] provided a guideline for keeping a laboratory notebook in computational biology that can also be applied to ELNs. Records must also include unintentional errors, as well as negative, unexpected, or conflicting results.

Over the past years, a tendency to promote reproducibility and transparency practices has been established among the scientific community [1]. In that respect, most scientific journals demand that researchers provide the raw data generated from their experiments [1,14]. To consolidate this trend, raw data availability should be mandatory in any research facility, regardless of the scientific discipline. OneNote’s data storage feature allows users to optimally collect raw files resulting from any scientific approach. However, storage of large datasets, such as high-quality images or sequencing files, will depend on Microsoft OneDrive or SharePoint storage plans. Alternatively, these datasets can be hyperlinked to internal or external hosting platforms. To avoid hosting-related accessibility issues, a representative file or low-quality image can be uploaded to OneNote along with a detailed description of these hyperlinked datasets. You can also consult the Ten Simple Rules paper by Hart and colleagues. on how to store digital data [17], especially rule 3: keep raw data raw.

In addition, protocols should contain detailed information to achieve a successful replication of the results by other researchers; for example, reference or lot number of any material or reagent should be listed. Plasmids, which are indispensable resources among researchers, should be meticulously described. Cloning experiments could therefore contain (1) a cloning strategy (e.g., Gibson cloning) describing all steps of the experimental approach; (2) results containing all positive and negative outcomes, including raw .seq and .ab1 files; and (3) a reconstruction of the final plasmid with its sequence, map, and features. Communications with collaborating researchers (e.g., e-mails or meeting highlights) having an impact on the outcomes of an experiment may also be documented.

## Data presentation

OneNote provides users with several tools to enhance data presentation. These tools are available at the Insert or Draw tabs. For instance, Microsoft Visio (OneNote *>* Insert *>* Diagram) is a diagramming application that could be used to sketch a protocol or a bioinformatic pipeline. Audio or video notes from lab meetings or experiments (e.g., mouse experiments) can also be recorded. OneNote’s optical character recognition (OCR) feature can be used to acquire text from old laboratory documents as well. OCR built-in capability converts the text from a picture, a scanned file, or a handwritten document into a machine-encoded, editable text.

OneNote’s compatibility with other Microsoft Office applications, such as PowerPoint or Excel, can give researchers an advantage for figure and table presentation. Graphs and tables constructed in these programs can be easily exported to OneNote. Figures—along with their legends, including graphs, diagrams, or imaging results (e.g., western blot outputs or microscopic images)—should be accurately labeled [18]. A legend should contain all information needed to understand and interpret the significance of a figure without reading the whole experiment. Tables must also be well organized with essential information, such as title, column headers, data, and footnotes.

A major advantage of ELNs over PLNs is their flexibility to record experiments continuously. Data generated from long experiments could be documented on a single page, separating each result by the exact date (OneNote > Insert > Time stamp) on which each part of the experiment was performed. There is no longer any need to create a page for each working day, as it is expected when using a PLN.

OneNote allows users to create multiple note containers (flexible bounding boxes) in one page [19]. Note containers, which can hold text, images, or files, behave as independent entries that can be moved around the page. This can lead to an unintentional overlap of these boxes and, consequently, to a superposition of text or images. To avoid this issue, all data must be included in a single note container. Table 1 summarizes all recommendations for data acquisition and presentation.

**Table 1:** Recommendations for data acquisition and presentation using Microsoft OneNote as an ELN.

Feature	Recommendation
**Acquisition**	All data resulting from any experiment, analysis, observation, etc., must be properly recorded without exception.
	Unintentional errors, as well as negative, unexpected, or conflicting results, should also be documented.
	All computational-related analyses along with their raw files, such as codes or scripts, should also be recorded.
	Raw data generated from any experimental approach could be uploaded within the ELN to promote reproducibility and transparency practices.
	Large datasets, such as high-quality images or sequencing files, can be hyperlinked to internal or external hosting platforms.
	Protocols and cloning experiments should contain detailed information (e.g., reference and lot number of any material or reagent) to assure reproducibility by other researchers.
	Important communications with collaborating researchers, such as e-mails or meeting highlights, may also be documented.
**Presentation**	Figures and tables should be self-explained with captions and detailed information.
	Improve data presentation by using OneNote tools available at “Insert” or “Draw” tabs (e.g., Microsoft Visio).
	Results from long experiments should be documented on a single page; there is no need to create a page for each working day.
	Use a single note container all along the experiment’s page to avoid unintentional overlapping of text or images.

Abbreviation: ELN, electronic lab notebook.

## Sharing

Pharmaceutical and academic research is habitually performed within a strong collaborative environment; in this regard, OneNote’s sharing features provide major advantages compared with PLNs. OneNote allows researchers to share their ELN among their team members and collaborators via a cloud-based platform such as Microsoft OneDrive or SharePoint [12]. In this manner, the experimental data are accessible from anywhere at any time, which is essential when an international collaboration has been established.

**Fig 2:**
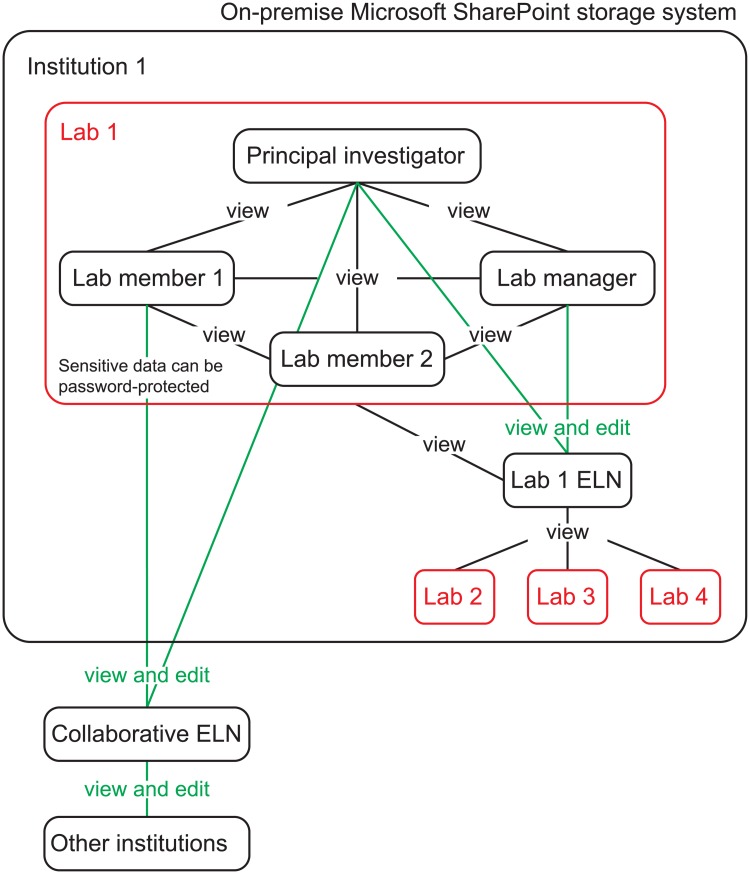
A schematic diagram presenting a OneNote ELN-sharing workflow. OneNote ELNs can be shared among lab members, laboratories, and institutions using two parameters: “can view” and “can edit and view.” ELN, electronic lab notebook.

Fig 2 shows an ELN-sharing workflow among institutions, laboratories, and lab members. Internally, ELNs could be shared among the members of the same laboratory under the mode “can view” to avoid accidental changes caused by any lab member. Legal documents, pages, or entire sections can be password-protected in order to secure confidential information (OneNote > Review > Password). In addition, we recommend sharing an entire ELN to other laboratories from the same institution to improve reproducible research practices and transparency [1]. This ELN could contain specific protocols, a list of sharable resources (e.g., primers, antibodies, cell lines, or chemical compounds), raw data, papers, or bioinformatic codes. All these data could be managed (“can view and edit” mode) by the principal investigator and/or the lab manager (Fig 2) and organized using OneNote group sections, sections, and pages. Because OneNote does not allow sharing of a specific section or a page [20], an entire ELN could be shared when an outside collaboration has been established. This ELN can be organized as described in Fig 1.

## Make sure to store, secure, and legalize your ELN correctly

ELN data storage and security are major concerns among researchers [12]. In this regard, we recommend to establish an on-premise Microsoft SharePoint storage system to prevent data security breaches related to cloud computing [12,21]. We also recommend backing up a full ELN to a OneNote package file (.onepkg); this single file contains the text, embedded files, audio, and video, similar to a .zip file. In addition, this on-premise system, under specific Microsoft SharePoint configurations, is able to provide customizable options to achieve compliance with the United States Food and Drug Administration’s Code of Federal Regulations Title 21 Part 11 (FDA 21 CFR Part 11). Part 11 is a US regulation that sets specifications on electronic records and electronic signatures (ERES). Furthermore, software development companies (e.g., Montrium and Paragon Solutions) provide services to reach compliance with EudraLex Volume 4 Annex 11, a European equivalent of the FDA 21 CFR Part 11 [12]. Regarding ERES, OneNote does not provide an option to electronically sign a note; however, completed experiments, protocols, etc., can be exported to .pdf format and be signed electronically. An ELN compliant with Part 11 or Annex 11 is considered as a legally accepted electronic document to protect researchers from legal matters such as fraud accusation or intellectual property theft [12].

## Go paperless and get connected

**Fig 3:**
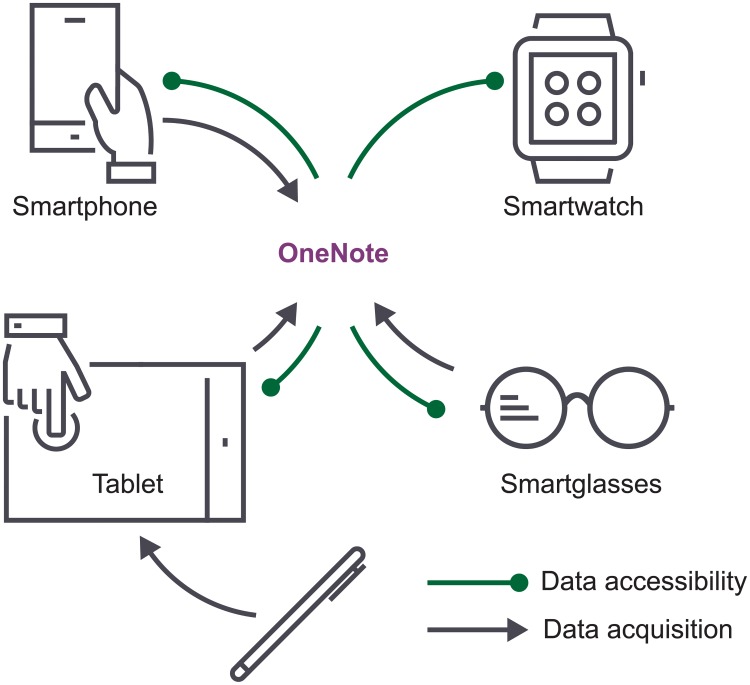
OneNote connectivity. Several external tools can be used to enhance OneNote ELN usage concerning data accessibility, acquisition, and presentation. ELN, electronic lab notebook.

Microsoft OneNote is able to interact with several external resources that can be used to improve data accessibility, acquisition, and presentation. Smartphones, tablets, and smartglasses can be used for audio and video recording, as well as for image capturing (Fig 3). The same devices, along with smartwatches, can also be used for data accessibility. For example, protocols can be displayed during experimentation and therefore minimize paper use and printing costs (Fig 3). In this regard, our survey-based study among 28 researchers using ELNs with tablet-based devices for a 3-month period showed that 67% of researchers think that tablets can substitute printed protocols [12]. Moreover, 80% of surveyed researchers think that tablets can improve ELN usage.

Wearable technology can also be implemented to improve data accessibility and visualization during OneNote usage in an experimental setting. For instance, protocols can be consulted through smartwatches or smartglasses. This represents a significant advantage when working in laboratory rooms or areas where PLNs are not allowed (e.g., cell culture or radioactive rooms). Both devices can also be used as substitutes for some bench equipment, such as chronometers or timers, and could provide researchers with other applications such as calculators, alarms, and reminders [12].

## OneNote ELN oversight and training

OneNote is a flexible tool that allows users to establish their own ELN design. This could lead to significant variations in ELN adoption and quality among lab members. We therefore recommend proper oversight and training of OneNote ELN usage and implementation. This should ensure the establishment of a general ELN format concerning protocols, templates, organization, etc. ELNs could be evaluated periodically by the principal investigator (e.g., approximately 4× per year) and be part of the employee’s appraisal process.

## References

[1] Iqbal SA, Wallach JD, Khoury MJ, Schully SD, Ioannidis JPA. Reproducible Research Practices and Transparency across the Biomedical Literature. PLoS Biol. 2016;14:e1002333 10.1371/journal.pbio.1002333 26726926 PMC4699702

[2] Collins FS, Tabak LA. Policy: NIH plans to enhance reproducibility. Nature. 2014;505:612–613. 10.1038/505612a 24482835 PMC4058759

[3] Kanza S, Willoughby C, Gibbins N, Whitby R, Frey JG, Erjavec J, et al Electronic lab notebooks: can they replace paper? J Cheminform. 2017;9:31 10.1186/s13321-017-0221-3 29086051 PMC5443717

[4] Guerrero S, López-Cortés A, Indacochea A, García-Cárdenas JM, Zambrano AK, Cabrera-Andrade A, et al Analysis of Racial/Ethnic Representation in Select Basic and Applied Cancer Research Studies. Sci Rep. 2018;8:13978 10.1038/s41598-018-32264-x 30228363 PMC6143551

[5] Wurth L, Papasaikas P, Olmeda D, Bley N, Calvo GT, Guerrero S, et al UNR/CSDE1 Drives a Post-transcriptional Program to Promote Melanoma Invasion and Metastasis. Cancer Cell. 2016;30:694–707. 10.1016/j.ccell.2016.10.004 27908735

[6] Guerrero S, Libre C, Batisse J, Mercenne G, Richer D, Laumond G, et al Translational regulation of APOBEC3G mRNA by Vif requires its 5’UTR and contributes to restoring HIV-1 infectivity. Sci Rep. 2016;6:39507 10.1038/srep39507 27996044 PMC5171582

[7] López-Cortés A, Paz-y-Miño C, Cabrera-Andrade A, Barigye SJ, Munteanu CR, González-Díaz H, et al Gene prioritization, communality analysis, networking and metabolic integrated pathway to better understand breast cancer pathogenesis. Sci Rep. 2018;8:16679 10.1038/s41598-018-35149-1 30420728 PMC6232116

[8] Batisse J, Guerrero SX, Bernacchi S, Richert L, Godet J, Goldschmidt V, et al APOBEC3G Impairs the Multimerization of the HIV-1 Vif Protein in Living Cells. J Virol. 2013;87:6492–6506. 10.1128/JVI.03494-12 23576497 PMC3648125

[9] Riley EM, Hattaway HZ, Felse PA. Implementation and use of cloud-based electronic lab notebook in a bioprocess engineering teaching laboratory. J Biol Eng. 2017;11:40 10.1186/s13036-017-0083-2 29201138 PMC5701295

[10] Kwok R. How to pick an electronic laboratory notebook. Nature. 2018;560:269–270. 10.1038/d41586-018-05895-3 30082695

[11] Bromfield Lee D. Implementation and Student Perceptions on Google Docs as an Electronic Laboratory Notebook in Organic Chemistry. J Chem Educ. 2018;95:1102–1111. 10.1021/acs.jchemed.7b00518

[12] Guerrero S, Dujardin G, Cabrera-Andrade A, Paz-y-Miño C, Indacochea A, Inglés-Ferrándiz M, et al Analysis and implementation of an electronic laboratory notebook in a biomedical research institute. PLoS ONE. 2016;11:eD160428 10.1371/journal.pone.0160428 27479083 PMC4968837

[13] Kazic T. Ten Simple Rules for Experiments’ Provenance. PLoS Comput Biol. 2015;11:e1004384 10.1371/journal.pcbi.1004384 26485673 PMC4619002

[14] Hoffman JI. Reproducibility: Archive computer code with raw data. Nature.2016;534:326. 10.1038/534326d 27306179

[15] Schreier AA, Wilson K, Resnik D. Academic research record-keeping: best practices for individuals, group leaders, and institutions. Acad Med. 2006;81:42–7. Available: http://www.ncbi.nlm.nih.gov/pubmed/16377817. 16377817 10.1097/00001888-200601000-00010 PMC3943904

[16] Schnell S. Ten Simple Rules for a Computational Biologist’s Laboratory Notebook. PLoS Comput Biol. 2015;11:e1004385 10.1371/journal.pcbi.1004385 26356732 PMC4565690

[17] Hart EM, Barmby P, LeBauer D, Michonneau F, Mount S, Mulrooney P, et al Ten Simple Rules for Digital Data Storage. PLoS Comput Biol. 2016;12:e1005097 10.1371/journal.pcbi.1005097 27764088 PMC5072699

[18] Rougier NP, Droettboom M, Bourne PE. Ten Simple Rules for Better Figures. PLoS Comput Biol. 2014;10:e1003833 10.1371/journal.pcbi.1003833 25210732 PMC4161295

[19] Microsoft [Internet]. Work with note containers. [cited 2019 Feb 4]. https://support.office.com/en-us/article/work-with-note-containers-2826d317-b67c-40cb-a6b7-1fdf58e4acbe.

[20] Microsoft [Internet]. Share a page of notes or an entire notebook from OneNote for Windows 10. [cited 2018 Nov 7]. https://support.office.com/en-us/article/share-a-page-of-notes-or-an-entire-notebook-from-onenote-for-windows-10-d4a74a14-44a3-411e-8fb5-06e73ddf047f.

[21] Ali M, Khan SU, Vasilakos A V. Security in cloud computing: Opportunities and challenges. Inf Sci (Ny). 2015;305:357–383. 10.1016/J.INS.2015.01.025

